# 

**Published:** 1903-06-06

**Authors:** 


### NOTICE TO CORRESPONDENTS.

All MSS., Letters, Books for Review, and other matters intended for the Editor should be addressed The Editor, "The Hospital," The Hospital Buildings, 28 & 29 Southampton Street, Strand, W.C.

The Editor cannot undertake to return rejected MS., even when accompanied by stamped directed envelope.

**Notice to Advertisers and Subscribers.**

All Advertisements, Orders for Copies of The Hospital, and Business Communications should be addressed to **THE MANAGER, (Not to the Editor),** The Hospital, 28 & 29 Southampton Street, Strand, London, W.C.

The Cost of Subscription, post free, is as follows.—Per week, 3½d.; per quarter, 3s. 9d.; per half-year, 7s. 6d.; per annum, 15s. Foreign and Colonial:—Per annum, 19s. 6d., payable, in all cases, in advance.

**NOTICE.—Vol. XXXIII. of "The Hospital" (October 1902 to March 1903) in handsome cloth binding, will shortly be on sale at the office of this Journal, price 7s. 6d. Cases for binding the Numbers contained in this Volume 1s. 6d. Index to Vol. XXXIII., 2½d., post free.**

**The monthly part for June is now ready. Price 1s., by post 1s. 3d.**

### BOOKS RECEIVED.

"Report of the Public Analyst of the City of Birmingham, 1902."
"Report on the Health of the City of Birmingham, 1902."

H. K. LEWIS.

"The Middlesex Hospital Reports of the Medical, Surgical, Obstetric, and Pathological Registrars, 1901."

BAILLIÈRE, TINDALL AND COX.

"Muco-Membranous Colitis." By Dr. Froussard.

HEALTH RESORTS BUREAU.

"Tenby."

J. WRIGHT AND CO., BRISTOL.

"Simple Rules for Preventing Infantile Complaints and Deaths among Infants." By J. T. C. Nash.
"The Pocket Therapist." By T. Stretch Dowse.

T. C. AND E. C. JACK.

"Breakfast and Savoury Dishes." By Florence B. Jack.
"Hot Puddings, Souffles, and Puddings." By Florence B. Jack.
"The Art of Cooking for Invalids." By Florence B. Jack.

JOHN BALE, SONS AND DANIELLSON.

"Squint." By C. Worth.

THE SCIENTIFIC PRESS, LIMITED.

"Burdett's Hospitals and Charities, 1903."

### THE STUDENT OF MEDICINE.

### APPOINTMENTS.

**H. G. Biddle**, M.R.C.S., L.R.C.P.Lond., appointed Medical Officer and Public Vaccinator for the St. Peter's District of the Isle of Thanet Union. **W. E. Bracey**, L.R.C.P.&S.Edin., L.F.P.S.Glasg., appointed Medical Officer and Public Vaccinator for the Wedmore District of the Axbridge Union. **Thos. P. Cann**, M.D., B.S. Durh., appointed Certifying Factory Surgeon for the Newhaven District, Sussex. **Daniel Patrick Coady**, F.R.C.S.I., L.R.C.P. &S.Edin., appointed Certifying Factory Surgeon for the Naas District, County Kildare. **F. Coleman**, M.R.C.S., L.R.C.P., L.D.S.Eng., appointed Dental Surgeon to the Metropolitan Hospital. **A. F. Constan**, M.B., Ch.B. Vict., appointed House Surgeon to the Manchester Royal Infirmary. **A. B. Cridland**, M.R.C.S., L.R.C.P., appointed House Surgeon to the Wolverhampton Eye Infirmary. **James Crooks**, M.D., C.M.Toronto, L.R.C.S. Edin., appointed Certifying Factory Surgeon for the Axminster District, Devonshire. **D. L. Dewar**, M.B., C.M.Glasg., appointed District Medical Officer of the Teesdale Union. **A. J. Edmonds**, M.B., Ch.B.Vict., appointed House Physician to the Manchester Royal Infirmary. **M. F. Ellis**, L.R.C.P.&S.Edin., L.F.P.S.Glasg., appointed District Medical Officer of the Holsworthy Union. **Thomas B. Fairclough**, L.R.C.P.&S.Edin., appointed Certifying Factory Surgeon for the Dewsbury District of the West Riding of Yorkshire. **Charles W. Forsyth**, M.R.C.S., L.R.C.P., appointed Assistant Medical Officer at St. Mary Islington Infirmary, Highgate Hill. **F. G. Gardner**, M.R.C.S.Eng., appointed Medical Officer of the Warwick Union Workhouse. **Thomas Gibson**, M.D.Edin., D.P.H.R.C.P.S.Lond., appointed Medical Officer of Health for Wakefield. **J. Strickland Goodall**, M.B.Lond., etc., appointed Lecturer on Physiology in the Medical School, Middlesex Hospital. **E. M. W. Hearn**, M.R.C.S.Eng., L.R.C.P.Lond., appointed Medical Superintendent of the Bolingbroke Hospital, Wandsworth Common, S.W. **John P. Henry**, M.D., B.Ch.Univ. Dublin, reappointed Oculist to the London School Board. **J. G. Jack**, M.B., Ch.B.Edin., appointed Certifying Factory Surgeon for the Falkland District, Fifeshire. **H. Carter Mactier**, B.A., M.B., B.Ch., B.A.O.Dubl. Univ., appointed Assistant Surgeon to the Wolverhampton Eye Infirmary. **William Meagher**, L.R.C.P.& S.I., appointed Certifying Factory Surgeon for the Ferbane District, King's County. **C. B. T. Musgrave**, M.D.Lond., appointed District Medical Officer of the Launcester Union and of the Tavistock Union. **Stewart Grant Ogilvy**, M.B., C.M.Edin., appointed Certifying Factory Surgeon for the Fauldhouse District of Linlithgow. **Hugh Prytherch**, L.R.C.P.Edin., M.R.C.S.Eng., appointed Certifying Factory Surgeon for the Beaumaris District, Anglesey. **J. S. Purdy**, M.B., C.M.Aberd., appointed House Surgeon to the London Male Lock Hospital. **P. H. Rainbird**, L.R.C.P.&S.Edin., appointed District Medical Officer of the Lincoln Union. **J. A. C. Ray**, M.B., Ch.B.Vict., appointed House Surgeon to the Manchester Royal Infirmary. **J. D. Rice**, M.B., B.Ch., R.U.I., appointed Medical Officer and Public Vaccinator for the New Eltham District of the Lewisham Union. **C. Simpson**, M.B., C.M.Aberd., appointed Certifying Factory Surgeon for the Towcester District, Northamptonshire. **D. D. Turner**, M.R.C.S., L.R.C.P.Lond., appointed Assistant to the Medical Superintendent of the Paddington Parish Infirmary. **E. Garney Wales**, M.A., M.B.Cantab., M.R.C.S., L.R.C.P., appointed Medical Officer of Health to the Urban District of Downham Market, Norfolk. **W. Cheyne Wilson**, M.D.Edin., appointed Honorary Physician to the Plymouth Public Dispensary. **M. S. Wood**, M.B., Ch.B.Vict., appointed House Physician to the Manchester Royal Infirmary. **Thos. Sanders Worboys**, M.R.C.S., L.R.C.P.Lond., appointed Certifying Factory Surgeon for the Winterton District, Lincolnshire.

### "The Hospital" Institutional Library.

"Hospitals and Asylums of the World." 4 Vols., with a Portfolio of Plans, complete £12 12s.

"Burdett's Hospitals and Charities, 1902." 5s.

"Burdett's Official Nursing Directory, 1903." 3s. net.

"Cottage Hospitals, General, Fever, and Convalescent." 10s. 6d.

"Uniform System of Accounts for Hospitals and Public Institutions." New Edition. 4s. net.

"Hospital Expenditure: The Commissariat." 2s. 6d.

"A Legal Handbook for the Use of Hospital Authorities." 2s. 6d.

"The Cottage Hospital Case Book or Register of Patients." 10s. and 12s. 6d.

"The Furnishing and Appliances of a Cottage." 6d.

All these are published by the SCIENTIFIC PRESS, Ltd., and may be obtained through any bookseller, or direct from the publishers, 28 and 29 Southampton Street, Strand, London, W.C.

### 1.—PRACTICAL HINTS ON DISTRICT NURSING.

By AMY HUGHES, late of the Central Training Home, Queen Victoria's Jubilee Institute for Nurses for the Sick Poor, and Lady Superintendent of Nurses, Bolton Union Workhouse.

Price **1s.** post free.

### 2.—THE ADVANTAGES AND PRIVILEGES OF A TRAINED DISTRICT NURSE AND THE DUTIES OF THE DISTRICT TOWARDS HER.

A useful little pamphlet.

By Sir HENRY BURDETT, K.C.B.

Price per copy **1½d.,** post free **2d**

### 3.—THE POCKET CASE-BOOK FOR DISTRICT AND PRIVATE NURSES.

By a PHYSICIAN.

Suitable size for pocket, cloth boards, **1s.** post free.

In handsome leather, gilt, **1s. 6d.** net, **1s. 7d.** post free.

### 4.—THE MIDWIVES' POCKET BOOK

(with Prefatory Chapter on the Midwives Bill).

A valuable little work.

By HONNOR MORTEN. Price **1s.** post free.

### 5.—ON PREPARATION FOR OPERATION IN PRIVATE HOUSES.

By a SURGEON. **6d.** post free.

### 6.—ART OF FEEDING THE INVALID.

By a MEDICAL PRACTITIONER and a LADY PROFESSOR OF COOKERY.

**1s. 6d.** post free.

---

*These books are obtainable of all Booksellers and are published by*
THE SCIENTIFIC PRESS, LIMITED, 28 & 29 Southampton Street, Strand, London,
*who will send their latest Catalogue of Nursing Text-books post free to any Nurse on application.*

